# Supplementary material for: Plasma Proteome Profiling to detect and avoid sample‐related biases in biomarker studies
Source: EMBO Mol Med. 2019 Sep 30;11(11):e10427. doi: 10.15252/emmm.201910427 (PMC6835559; doi:10.15252/emmm.201910427)
Supplement: Supplementary file 1 — Appendix [file EMMM-11-e10427-s001.pdf]

Table of content

Appendix Figure S1: Number of identified proteins per sample type for the 20 study participants. (Page 2)

Appendix Figure S2: Correlations of the main blood fractions. (Page 2)

Appendix Figure S3: Correlation of the quality marker panels to plasma protein levels in the weight loss study. (Page 3)

Appendix Figure S4: Levels of the top four proteins of each panel across all samples in the weight loss study. (Page 4)

Appendix Figure S5: Erythrocyte and coagulation marker in the global correlation map. (Page 5)

Appendix Figure S6: Cross-comparison of serum and different plasma species. (Page 6)

Appendix Figure S7: Comparison of pull and vacuum sampling systems. (Page 6)

Appendix Figure S8: Comparison of gel containing and no gel containing sampling systems. (Page 7)

Appendix Figure S9: Distribution of platelet proteins in different plasma volume fractions after centrifugation. (Page 8)

Appendix Figure S10: Distribution of erythrocyte proteins in different plasma volume fractions after centrifugation. (Page 9)

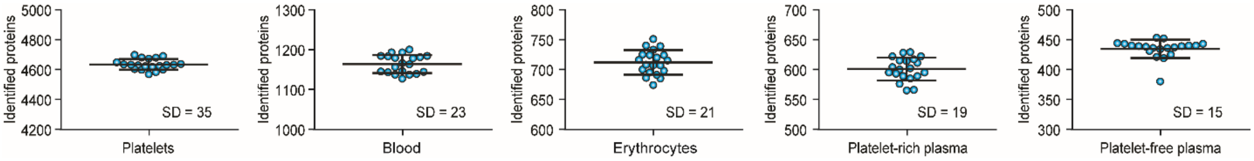

Appendix Figure S1: Number of identified proteins per sample type for the 20 study participants. The number of identified proteins is shown for all individuals. The whiskers indicate the standard deviation (SD) and the mean is also indicated.

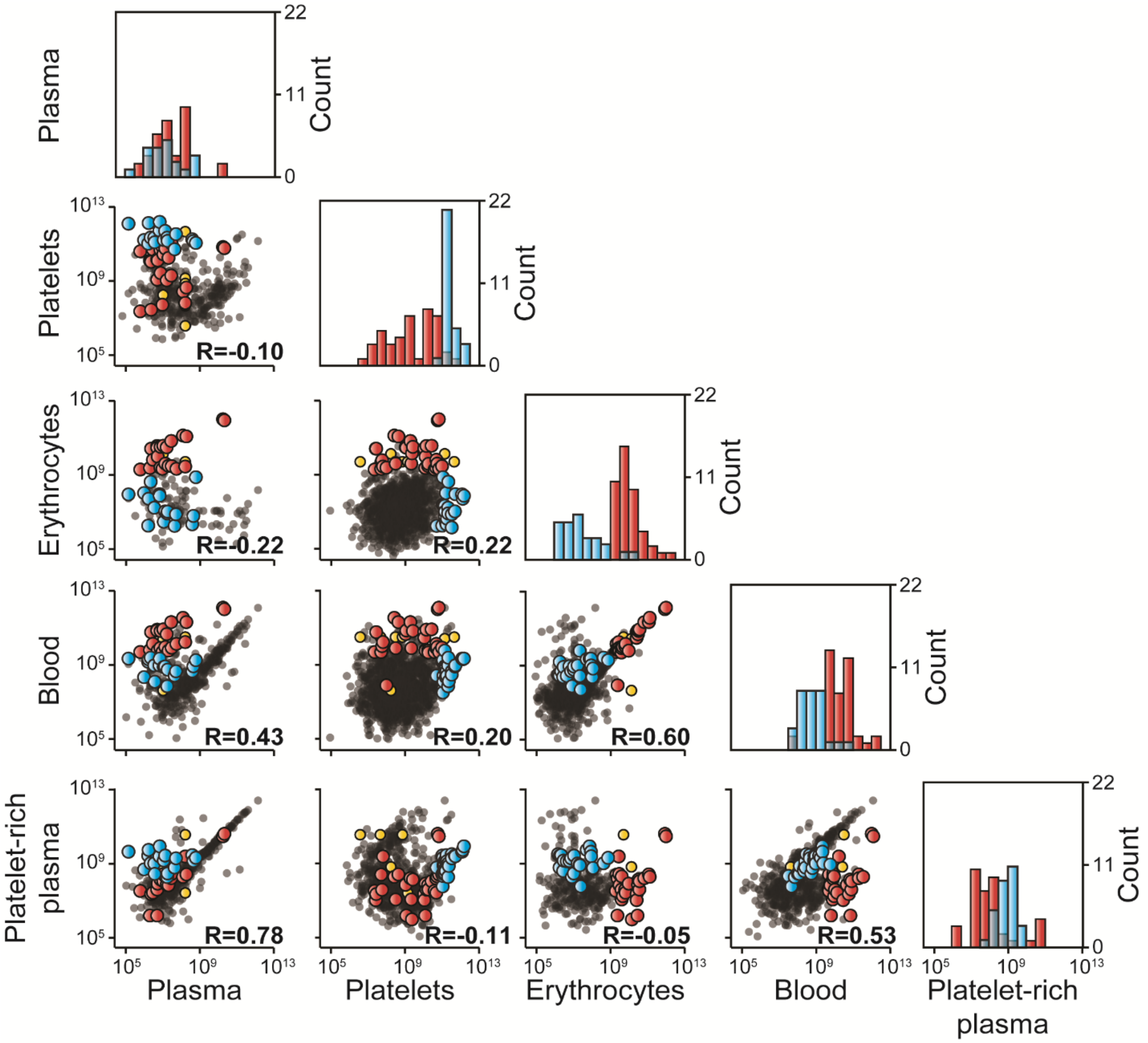

Appendix Figure S2: Correlation of the main blood fractions. Correlation of the median proteomes of whole blood, erythrocytes, platelets, platelet-rich plasma and platelet-free plasma from 20 individuals. The 30 highest abundant proteins of erythrocytes and platelets are highlighted in red and blue, respectively. Proteins highlighted in yellow overlap between the top 30 proteins in erythrocytes and platelets. The histograms show the distribution of both marker panels over the abundance range of the plasma, platelet, erythrocyte, blood and platelet-rich plasma proteome, respectively.

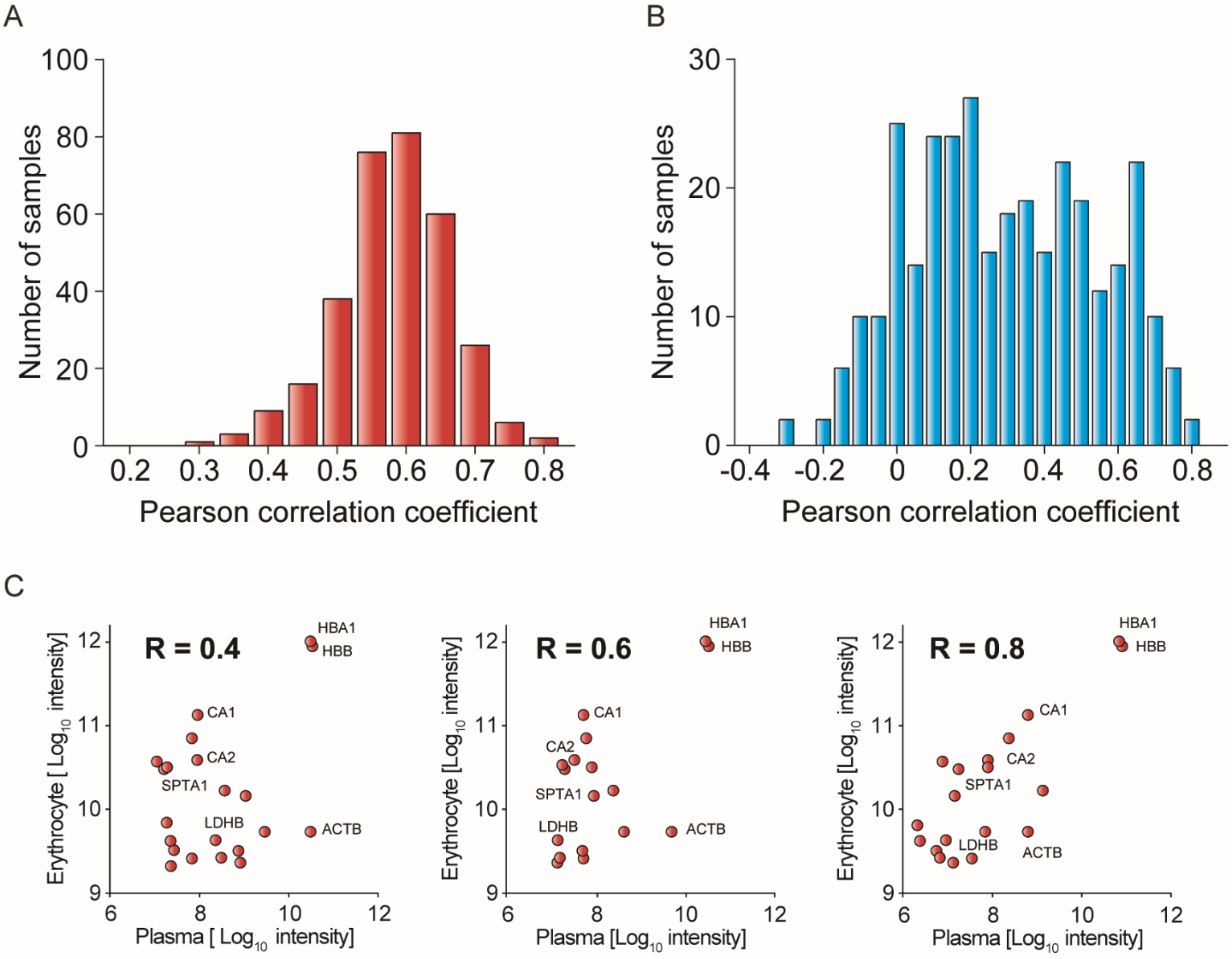

Appendix Figure S3: Correlation of the quality marker panels to plasma protein levels in the weight loss study. (A) Histogram of Pearson correlation coefficients calculated between the reference cohort and the plasma samples in the weight loss study for the erythrocyte quality marker panel (Geyer et al, 2016). (B) Distribution of the Pearson correlation coefficients for the platelet panel. (C) Exemplified correlations for the erythrocyte panel in three samples.

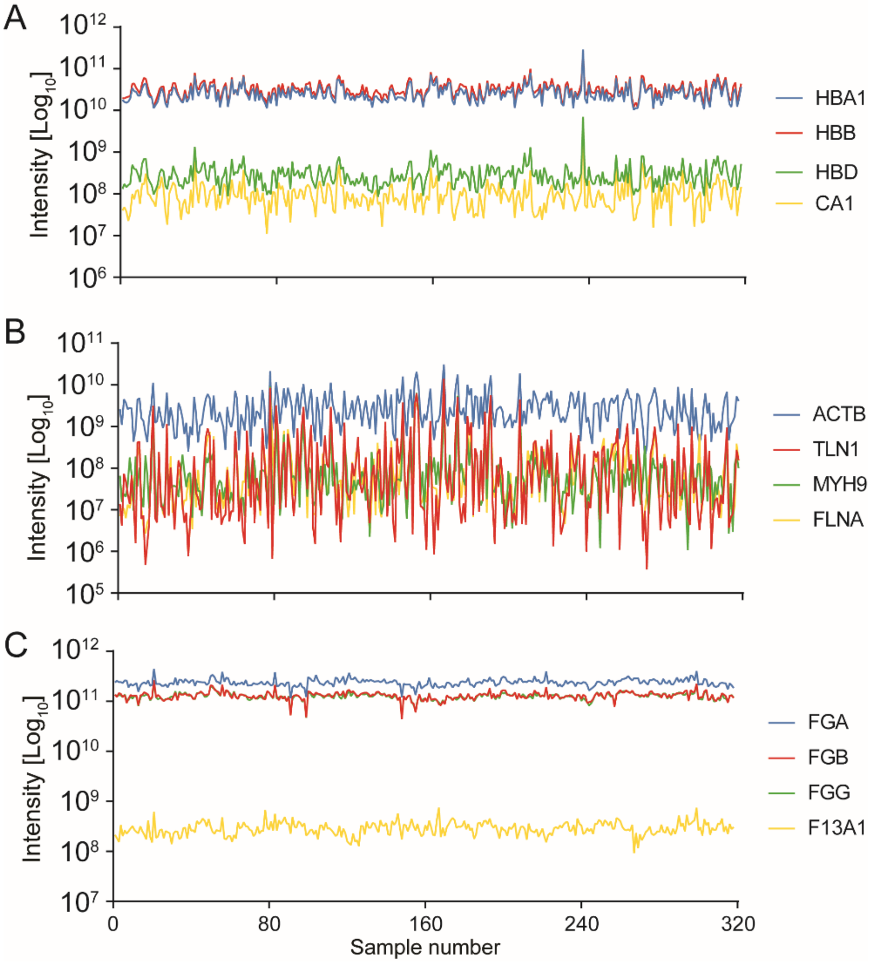

Appendix Figure S4: Levels of the top four proteins of each panel across all samples in the weight loss study. (A) Intensities of the four highest abundant erythrocyte specific proteins for all samples. (B) Intensities of the four highest abundant platelet proteins. (C) Intensities of the four most significantly regulated coagulation markers.

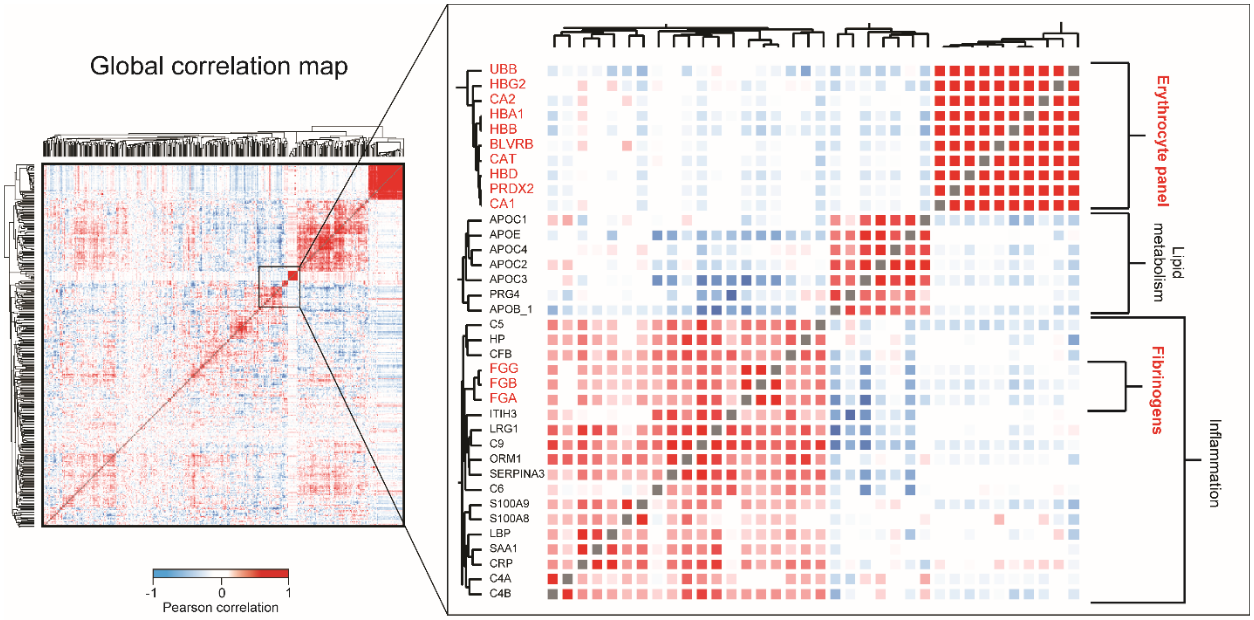

Appendix Figure S5: Erythrocyte and coagulation marker in the global correlation map. The global correlation map is shown on the left and the magnified inset shows three clusters of correlating proteins. The erythrocyte panel and the fibrinogens are highlighted in red. The color-code for the Pearson correlation coefficient is indicated.

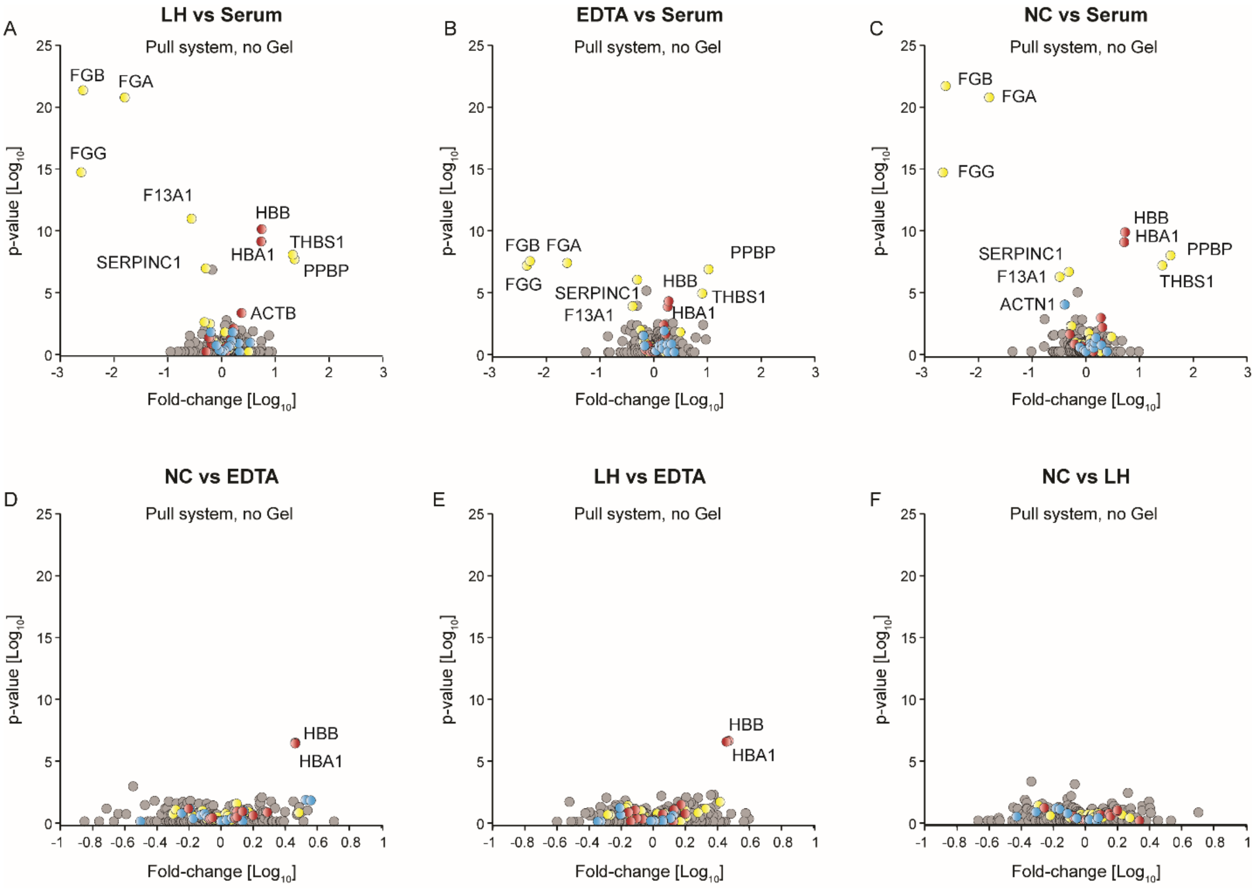

Appendix Figure S6: Cross-comparison of serum and different plasma species. (A-F) Pairwise comparison between two of the four species is indicated above the volcano plots. The x-axes reflect the log10-fold changes and the y-axes the t-test p-values. Proteins with statistically significant different protein levels between the comparisons are highlighted with labeled names. Erythrocyte, platelet and coagulation panel markers are highlighted in red, blue and yellow, respectively.

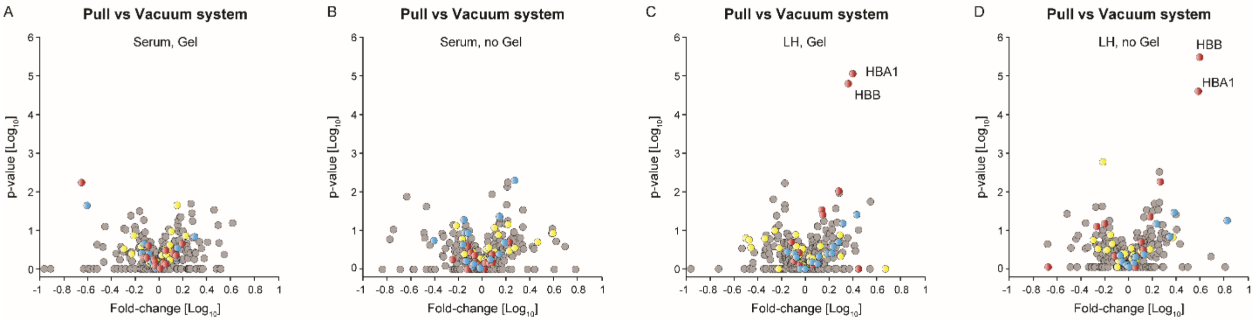

Appendix Figure S7: Comparison of pull and vacuum sampling systems. (A-D) Comparisons of piston plunger and vacuum sampling systems for serum and lithium heparin plasma with and without gel plugs. The x-axes reflect the log10-fold changes and the y-axes the t-test p-values. Proteins with statistically significant different protein levels between the comparisons are highlighted with labeled names. Erythrocyte, platelet and coagulation panel markers are highlighted in red, blue and yellow, respectively.

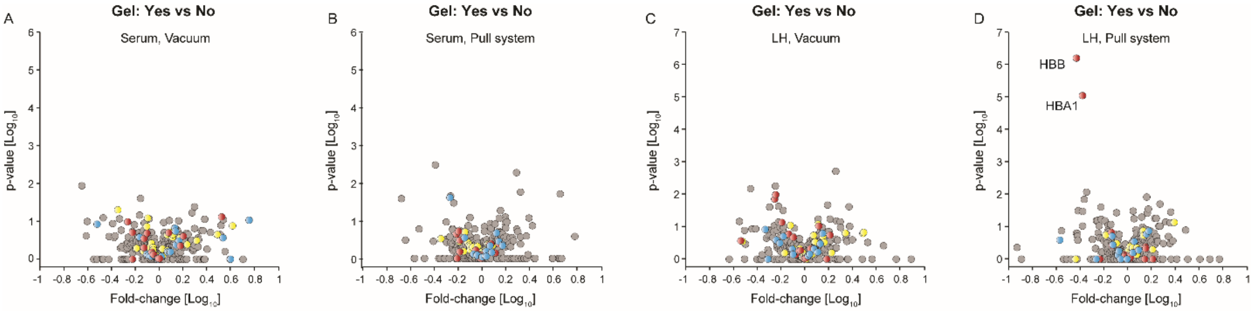

Appendix Figure S8: Comparison of gel containing and no gel containing sampling systems. (A-D) The comparisons of sampling systems with or without gel plugs for serum and lithium heparin plasma with pull or vacuum sampling systems. The x-axes reflect the log10-fold changes and the y-axes the t-test p-values. Proteins with statistically significant different protein levels between the comparisons are highlighted with labeled names. Erythrocyte, platelet and coagulation panel markers are highlighted in red, blue and yellow, respectively.

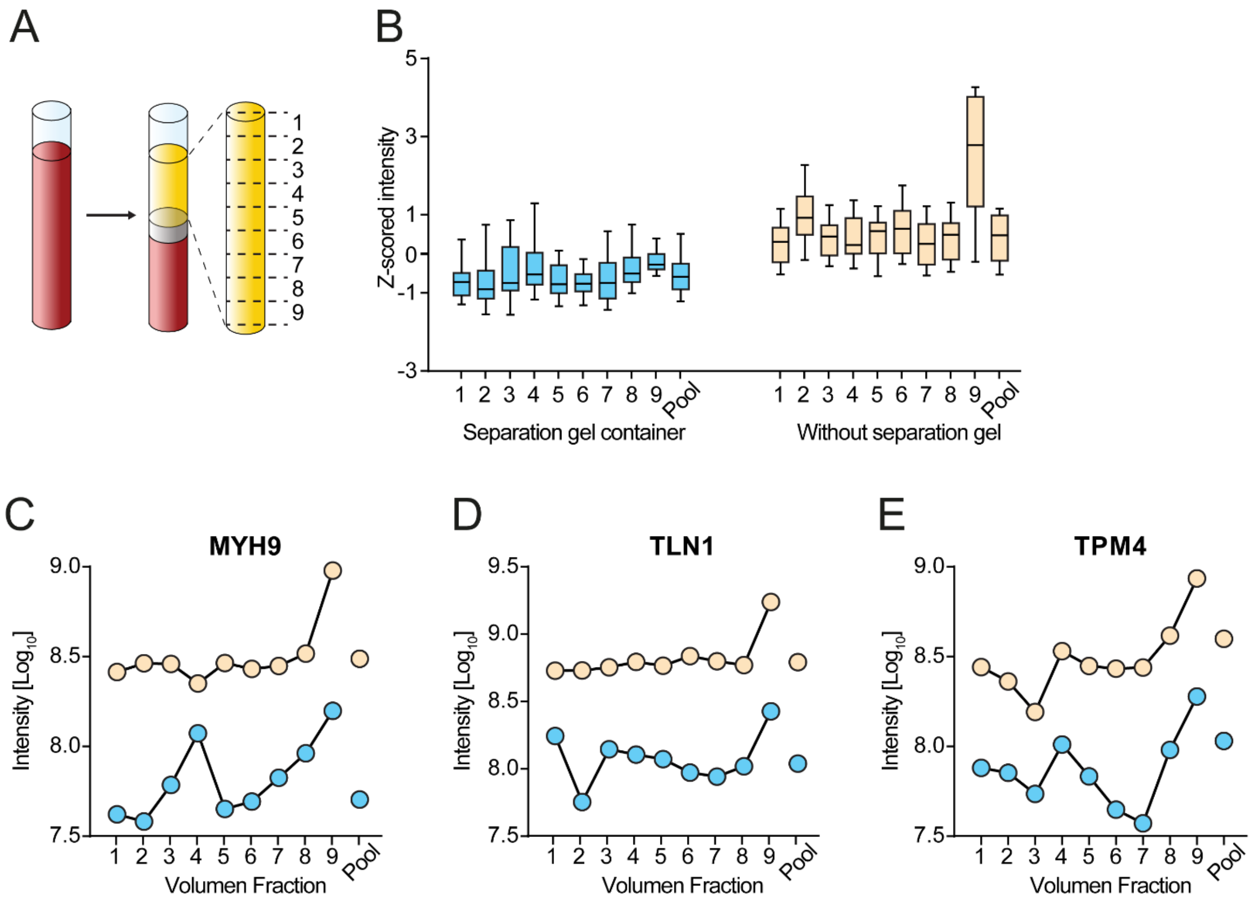

Appendix Figure S9: Distribution of platelet proteins in different plasma volume fractions after centrifugation. (A) Plasma from nine different layers were harvested starting from the top after centrifugation to the top above the buffy coat in 500 µl steps. (B) The boxplots indicate the Z-scores of 27 of the top 30 platelet proteins that were quantified with at least 50% valid values in this experiment for the volume fractions 1-9 and the pool of all layers. The whiskers indicate the 10-90% quartile and the horizontal line within the boxplots is the median. (C-E) Examples for three platelet proteins for the three plasma collection protocols with intensity values, illustrating the changes within protocol and the volume fractions. Data points of the centrifugation container with and without a gel plug are color coded in blue and beige, respectively.

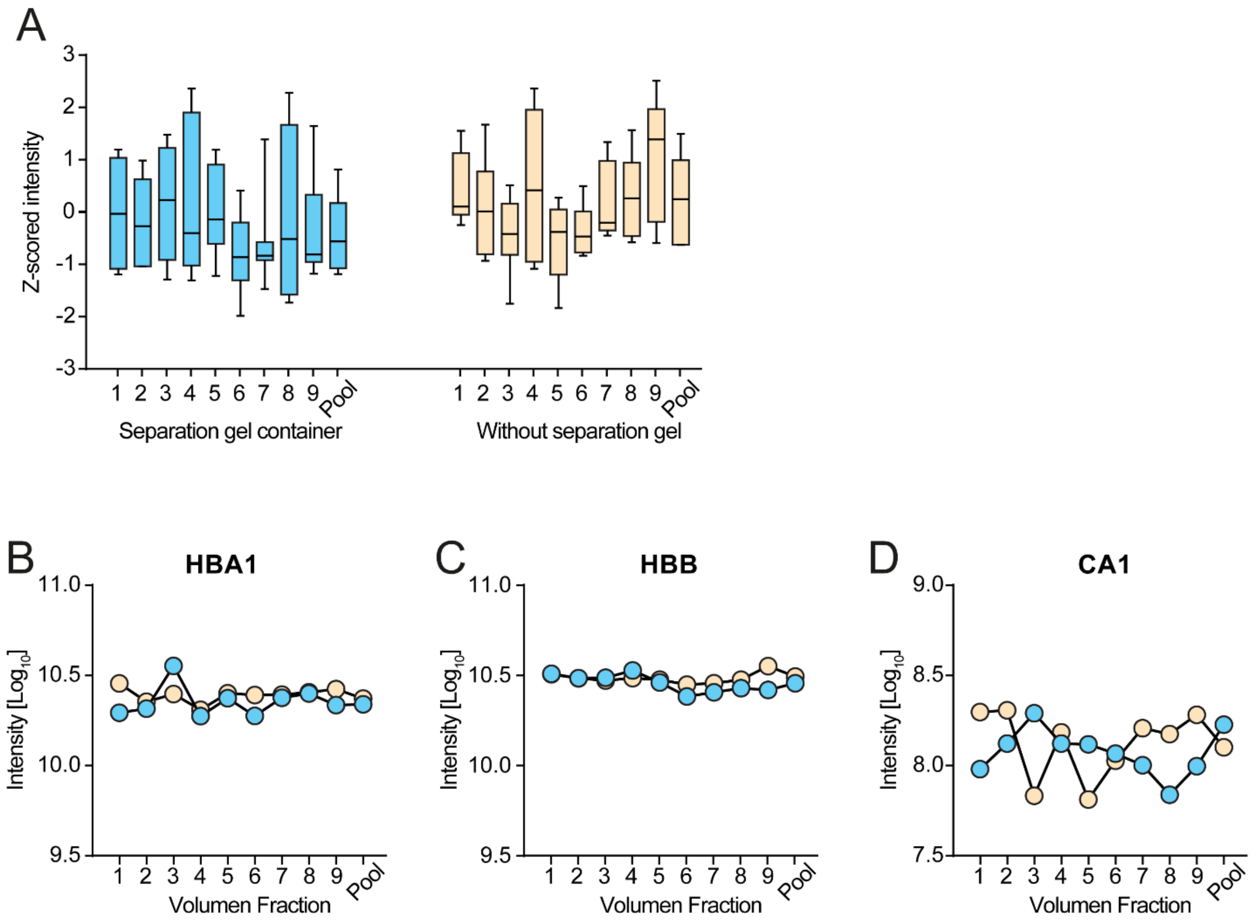

Appendix Figure S10: Distribution of erythrocyte proteins in different plasma volume fractions after centrifugation. (A) Volume fractions 1-9 and the pool of all layers for three different plasma collection protocols. The boxplots indicate the Z-scores of 7 of the top 30 erythrocyte proteins that were quantified with at least 50% valid values in this experiment. The whiskers indicate the 10-90% quartile and the horizontal line within the boxplots is the median. (C-E) Examples for three erythrocyte proteins for the three plasma collection protocols with intensity values, illustrating the changes within protocol and the volume fractions. The color code is according to panel B indicating the three protocols.
